# Composite Structured M/Ce_0.75_Zr_0.25_O_2_/Al_2_O_3_/FeCrAl (M = Pt, Rh, and Ru) Catalysts for Propane and n-Butane Reforming to Syngas

**DOI:** 10.3390/ma15207336

**Published:** 2022-10-20

**Authors:** Natalia Ruban, Vladimir Rogozhnikov, Sergey Zazhigalov, Andrey Zagoruiko, Vyacheslav Emelyanov, Pavel Snytnikov, Vladimir Sobyanin, Dmitriy Potemkin

**Affiliations:** 1 Boreskov Institute of Catalysis, Pr. Akademika Lavrentieva, 5, 630090 Novosibirsk, Russia; 2 Faculty of Natural Science, Novosibirsk State University, Pirogova St., 2, 630090 Novosibirsk, Russia; 3 Department of Physical and Colloid Chemistry, Faculty of Chemical Technology and Ecology, Gubkin Russian State University of Oil and Gas, Leninsky Pr., 65, 119991 Moscow, Russia; 4 Nikolaev Institute of Inorganic Chemistry, Pr. Akademika Lavrentieva, 3, 630090 Novosibirsk, Russia; 5 Department of Environmental Engineering, Novosibirsk State Technical University, Karl Marx Pr., 20, 630073 Novosibirsk, Russia

**Keywords:** steam reforming, autothermal reforming, propane, butane, structured catalysts

## Abstract

Here, we report the preparation, characterization, and performance of reforming propane and n-butane into a syngas of composite structured M/Ce_0.75_Zr_0.25_O_2_/Al_2_O_3_/FeCrAl (M = 0.46 wt.% Pt, 0.24 wt.% Rh, and 0.24 wt.% Ru) catalysts. The catalysts are composed of a high-heat-conducting FeCrAl block with preset geometry, with a surface nearly totally covered by θ-Al_2_O_3_. Afterwards, a layer of ceria–zirconia mixed oxide was deposited. The formed oxide coating was used as a support for 2–3 nm sized Pt, Rh, or Ru nanoparticles. The performance of the catalysts in propane steam reforming decreased in the order of Rh ≈ Ru > Pt. The reformates obtained in the propane steam reforming over Rh- and Ru/Ce_0.75_Zr_0.25_O_2_/Al_2_O_3_/FeCrAl at 600 °C and GHSV = 8300 h^−1^ contained 65.2 and 62.4 vol.% of H_2_, respectively, and can be used as a fuel for solid oxide fuel cells. In the oxidative steam reforming of propane at 700 °C and GHSV= 17,000 h^−1^, the activities of the Rh- and Pt-based catalysts were similar and the compositions of the outlet gas mixtures were quite close to equilibrium in both cases. Increasing the reagent flow rate to 25,600 h^−1^ showed stability of the Rh/Ce_0.75_Zr_0.25_O_2_/Al_2_O_3_/FeCrAl performance, whereas the Pt/Ce_0.75_Zr_0.25_O_2_/Al_2_O_3_/FeCrAl activity decreased. A mathematical model considering the velocity field, mass balance, pressure, and temperature distribution, as well as the reaction kinetics, was suggested for the propane steam and oxidative steam reforming over the Pt- and Rh/Ce_0.75_Zr_0.25_O_2_/Al_2_O_3_/FeCrAl catalysts. The model well described the experimental results.

## 1. Introduction

The most important global issues such as climate change and pollution of the environment focus the scientific society’s attention on the development of ecology-friendly energy production. Although in some developed countries (i.e., Germany and Denmark), the share of energy generated by renewable sources is increasing in a stable tendency [1], it is predicted that fossil fuels’ share in total energy supply will be significant for at least 20 more years. For these reasons, the development of more efficient and sustainable ways to generate energy from fossil fuels is an important and quite immediate task.

Fuel cells are considered as a perspective technology for efficient electricity generation directly from the energy of chemical bonds. The most convenient fuel for fuel cells is hydrogen. The use of hydrogen as a fuel for fuel cells leads to the stable working cycle of power generators and makes the technology environmentally friendly. On the other hand, at this moment, logistical problems associated with hydrogen are major restraining factors for fuel cell technologies. Nowadays, the main sources of hydrogen are fossil fuels such as natural gas and petrochemicals [2]. Therefore, hydrogen production from fossil fuels immediately before the working cycle of fuel cell-based power generators can solve the logistical problems and provide stable work for the fuel cells.

In the cases of logistics and energy density, the most attractive fuels are the liquid products of the oil industry: gasoline, diesel, or jet fuel. However, the composition of these types of fuel is complicated and strongly depends on the region and manufacturer, which can negatively impact the stability of power generation. Because of these reasons, liquified petroleum gases (LPGs), primarily consisting of propane and butane, may be considered as golden means. On the one hand, contrariwise liquid fuels in the composition of LPGs are minimally converted components, especially aromatic compounds. Further, LPGs are preferable to natural gas because of the liquefaction opportunity in quite soft conditions (*p* > 10 bar) [3]. In addition, synthetic LPGs can be easily obtained from “green” hydrogen and captured CO_2_ via the hydrogenation reaction, closing the carbon-free energy cycle. Hence, the development of highly efficient catalysts for LPG reforming is an important task for fuel cell energy production.

The development of fuel cell technologies has created a special demand for mobile and local power stations. The current industrial technologies of hydrogen generation from hydrocarbons are not optimal at these types of stations [4]. First, commonly used steam reforming is an endothermal process; therefore, the technology requires a constant external heat input. A combination of endothermal steam and exothermal partial hydrocarbon oxidation, namely, oxidative steam reforming (OSR), can provide high hydrogen productivity and decrease heat input. For the effective use of OSR benefits, it is important to provide heat transfer from the short front zone of the catalyst, where exothermal oxidation takes place, to the zone of steam oxidation. Structured catalysts formed from metal alloys have a level high thermal conductivity [5]. Moreover, it has been shown that using structured forms of catalysts can improve the efficiency of hydrocarbon catalytic reforming and decrease the overall cost of the process [6,7,8,9].

In the reforming processes, Ni-based catalysts are often used because of their rather good efficiency and low cost [10]. However, the impact of the standard problem of coke formation on the Ni-based catalyst surfaces [11] increases in this condition, whereas regeneration is undesirable. For medium-scale local or mobile power stations applying expensive, but more stable and efficient, noble metal-based catalysts may be a reasonable solution. According to data collected by the scientific community, the most active catalysts of C_2+_-hydrocarbon reforming are those based on Rh, Ru, Ir, and Pt [12,13,14]. Information about the compositions of efficient catalysts can be successfully used for the development of new catalytic system preparation methods [15], and in that work, we used information about the most active granular catalysts for designing structured catalysts.

Previously, we demonstrated a high-activity, Rh-based structured catalyst in the autothermal reforming of gasoline, diesel, and biodiesel [16,17]. It was found that the presence of aromatic compounds in the liquid fuel composition was associated with the decreasing efficiency of the catalytic reforming over the Rh-based structured catalyst. The composition of LPGs is more attractive because this type of fuel primarily consists of C_3_-C_4_ alkanes and does not contain aromatic compounds. In this work, we focused on the investigation of Rh-based structured catalyst activity in the steam and autothermal reforming of propane and n-butane as model compounds of LPGs. Ru- and Pt-based structured catalysts were prepared and tested with the fundamental goal of comparing the noble metal-based catalysts’ molar activities in LPG reforming. In addition, this research was conducted for economic reasons: the extremely high price of Rh motivated us to search for less expensive catalysts with suitable activities.

## 2. Materials and Methods

### 2.1. Preparation of the Structured Catalysts

**Figure 1:**
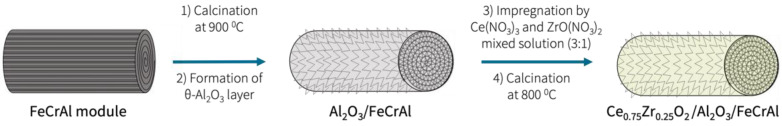
Scheme of the Ce_0.75_Zr_0.25_O_2_/Al_2_O_3_/FeCrAl preparation.

The preparation procedure of the structured catalysts started with the structured support formation. In the first step of the support preparation, a structured FeCrAl module (diameter = 18 mm and length = 60 mm) was formed from wire mesh of Fe, Cr, and Al alloy (NPO Souznichrom Inc., Moscow, Russia) and calcinated. In the next synthetic step, the structured FeCrAl module was covered by an θ-Al_2_O_3_ layer, according to reported technique in [18]. Then, on the θ-Al_2_O_3_/FeCrAl surface, a layer of Ce and Zr mixed oxide was formed. The preparation procedure included the 7-step impregnation of the θ-Al_2_O_3_/FeCrAl by a Ce(NO_3_)_3_ (AO Reahim, LLC, Moscow, Russia, 98% purity) and ZrO(NO_3_)_2_ (Interhim, LLC, Saint-Petersburg, Russia, 98% purity) mixed solution (the Ce:Zr molar ratio was 3:1). After each impregnation, the structured support was calcinated at 800 °C for 5 min and again for 30 min after the final impregnation. The structured support Ce_0.75_Zr_0.25_O_2_/θ-Al_2_O_3_/FeCrAl was formed. The preparation procedure of the structured support was similar for the Rh-, Ru-, and Pt-based structured catalysts and is described schematically in Figure 1.

The goal of this work was a comparison activity per 1 metal atom catalyst with an equimolar content of noble metal (M/CZA/FeCrAl, M = Rh, Pt, and Ru). For the Rh- and Pt-based structured catalysts, a sorption–hydrolytic preparation technique was used. According to the preparation procedure, a metal hydroxide was deposited on the structured support surface from the solution consisting of the noble metal precursor (RhCl_3_*4H_2_O and K_2_PtCl_4,_ respectively) and Na_2_CO_3_. The preparation procedure of the Ru/CZA/FeCrAl included impregnation of the structured support by a hot solution of the *fac*-[RuNO(NH_3_)_2_(NO_3_)_3_] precursor. The Ru-precursor was obtained from Ru(OH)Cl_3_ (Krastsvetmet, JSC, Krasnoyarsk, Russia, 99% purity), according to the 3-step technique described in [19,20,21]. Afterward, the precursor fixation catalysts were regenerated in the N_2_-H_2_ gas mixture at 750 °C.

### 2.2. Catalysts Characterization

Pieces of the structured FeCrAl module and the Al_2_O_3_/FeCrAl were separated using shears and investigated by an XRD technique. The analysis was carried out on an Arl X’Tra (Thermo Fisher Scientific, Waltham, MA, USA). CuKα radiation was used. Measurements were carried out in the 2θ range from 20 to 70° (step = 0.05°). The signal accumulation time was 5 s. For the XRD analysis of the Ce and Zr mixed oxide, a small piece of support coating was mechanically separated from the structured support. The analysis was carried out on diffractometer Bruker D8 Advance (Bruker, Karlsruhe, Germany) in CuKα radiation mode (λ = 1.5418 Å). Bragg–Brentano focusing was used. The measurements were carried out in the 2θ range of 15–75° (step = 0.05°). The signal accumulation time was 3 s. The analysis of the diffraction pictures was carried out using the ICDD PDF-2 database.

The structured catalysts were studied using transmission electron microscopy. Samples of the catalytic coating were mechanically separated for the investigations. The studies of the samples were carried out using a Themis Z electron microscope (Thermo Fisher Scientific).

The Rh/CZA/FeCrAl catalyst was investigated by temperature-programmed oxidation (TPO) method after tests in the propane and n-butane steam reforming. A quartz tubular reactor was used for the TPO experiments. The oxidation processes were carried out in a mixture of O_2_ and He (6 vol.% and 94 vol.%, respectively). A QMC-200 mass spectrometer was used for the registration of the CO_2_ concentration. The sensitivity of the measurements was 0.05 mg of carbon.

### 2.3. Catalytic Activity Investigations

Investigations of the catalytic activity were carried out in fixed-bed flow reactor. The steam and oxidative steam reforming were carried out at atmospheric pressure. An external steam generator was used for superheating the water to 150 °C. In the oxidative steam reforming steam, the fuel and air were mixed immediately before the reaction zone. The reactor was preliminarily heated up to 600 °C and 700 °C in a nitrogen flow before the steam and oxidative steam reforming, respectively.

The steam reforming of propane and n-butane was carried out at H_2_O/C = 3. In the oxidative steam reforming of the fuels, the H_2_O/C and O_2_/C ratios were fixed at 2.5 and 0.5, respectively. The reaction temperature was measured in the end zone of the catalyst.

Compositions of the obtained gas mixtures were analyzed using gas chromatography. For the analysis, a GC-1000 gas chromatograph (Chromos, Dzerzhinsk, Russia) was used. A thermal conductivity detector (TCD) and a flame ionization detector (FID) of the GC-1000 chromatograph allowed for analysis of the N_2_, H_2_, and carbon-containing components at the same time.

As experimental controls, an empty reactor and a reactor with a structured support Ce_0.75_Zr_0.25_O_2_/Al_2_O_3_/FeCrAl was heated up to 600 °C, and a propane–water mixture was added to the reaction zone. In both cases, the outlet gas mixture primarily consisted of propane (the water was separated before the analysis), whereas the hydrogen concentrations were lower than 2% in both cases.

## 3. Results

### 3.1. Characterization of the M/CZA/FeCrAl

**Figure 2:**
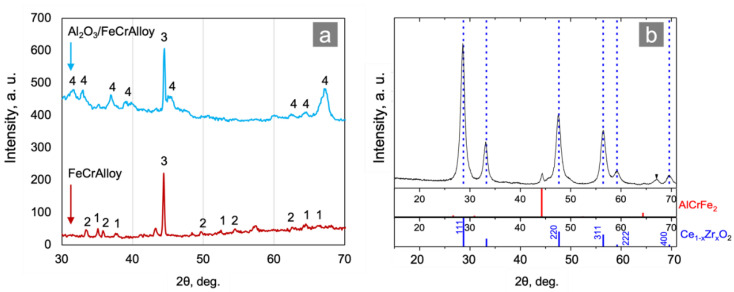
XRD patterns of the Al_2_O_3_/FeCrAl and FeCrAl: (**a**) 1—reflexes of α-Al_2_O_3_, 2—Fe and Cr (oxides), 3—Fe, Cr (metals), and 4—θ-Al_2_O_3_; and (**b**) a sample of the supporting coating.

**Figure 3:**
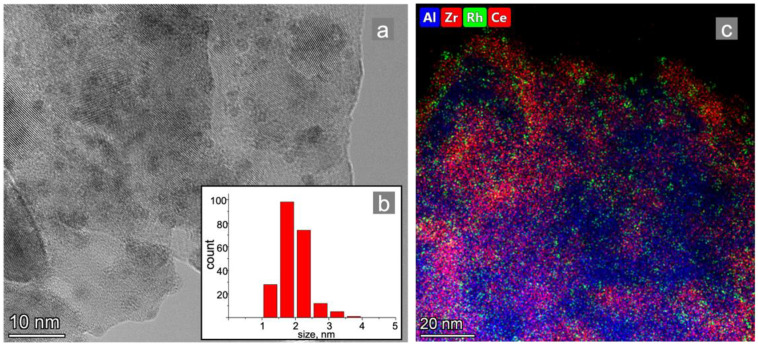
TEM images: (**a**) element distribution mapping of the (**c**) Rh/CZA/FeCrAl catalytic coating and (**b**) Rh particle size distribution.

**Figure 4:**
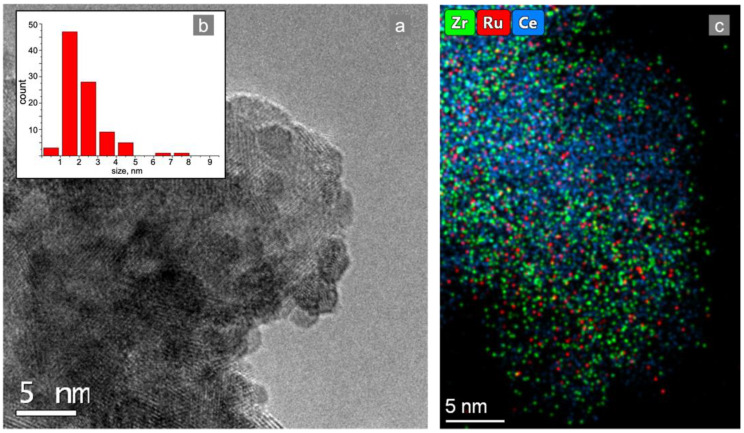
TEM images: (**a**) element distribution mapping of the (**c**) Ru/CZA/FeCrAl catalytic coating and (**b**) Ru particle size distribution.

The Ce_0.75_Zr_0.25_O_2_/Al_2_O_3_/FeCrAl structured supports were prepared and characterized using the XRD method after each synthetic step. As seen in Figure 2a, the FeCrAl surface was almost totally covered by θ-Al_2_O_3_ after the second synthetic step. The XRD data obtained for the sample of coating after the third synthetic stage (Figure 2b) proved that the support presented with a Ce_1–x_Zr_x_O_2_ mixed oxide with a fluorite-type structure. According to preparation procedure, the Ce:Zr molar ratio in the precursor solution was 3:1 therefore the average composition of the support that can be described by Ce_0.75_Zr_0.25_O_2_. As the preparation procedure of the Ce_1–x_Zr_x_O_2_ mixed oxide was non-selective, mixed oxide was represented by the number of solid solutions with a variable composition. It was also confirmed by the XRD (Figure 2b) and HAADF-STEM (Figure 3c and Figure 4c) analyses.

The support structure is stable in the condition of metal deposition, according to the literature data and our previous research [22]. A sample of the catalytic coating of the as-prepared Rh/CZA/FeCrAl was mechanically separated and studied using transmission electron microscopy (TEM). According to the TEM data (Figure 3a) and element distribution mapping (Figure 3c), the Rh particles were evenly distributed on the support surface. The average particle size was 2 nm (Figure 3b).

For the preparation of the Ru/CZA/FeCrAl catalyst, an impregnation method was used. One of the common problems with this method is the agglomerates formation of active metal particles. In this work, the support was impregnated by the specific precursor of Ru with the goals of preventing agglomerates formation and simultaneously simplifying the structured catalyst preparation procedure. A sample of the Ru/CZA/FeCrAl catalytic coating was investigated by the TEM technique. As seen in Figure 4b, the average Ru particle size was 2.3 nm and only small quantities of 6–8 nm-sized particles were found.

### 3.2. Propane Steam Reforming over the M/CZA/FeCrAl

**Figure 5:**
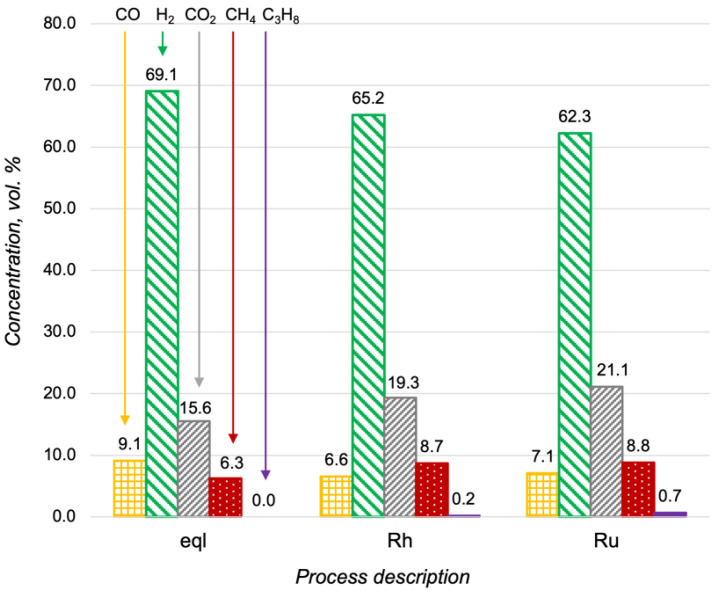
Product distribution in the propane steam reforming at 600 °C, with H_2_O/C = 3 and a flow rate of 8300 h^−1^, in a thermodynamic equilibrium (eql), over the Rh/CZA/FeCrAl (Rh), and over the Ru/CZA/FeCrAl (Ru).

Oxidative steam reforming (OSR) is a combination of the partial and steam oxidation of hydrocarbons. Steam reforming proceeds more slowly than partial oxidation and the activity of the catalyst in this process is critically important. As the first stage of investigation, the propane steam reforming over the Ru-, Pt- and Rh/CZA/FeCrAl was studied. The results of the Ru- and Rh/CZA/FeCrAl catalytic activity investigations at 600 °C, with H_2_O/C = 3 and a flow rate of 8300 h^−1^, are presented in Figure 5, where compositions of the reaction products were compared with product distribution in thermodynamic equilibrium. As seen in Figure 5, the product distributions were quite close to an equilibrium condition in both cases, but the concentration of unconverted fuel was higher in the case of the Ru/CZA/FeCrAl. The obtained data for the Pt/CZA/FeCrAl was not presented in Figure 5 because of the low activity of the catalyst—the propane conversion was less than 10% in the investigated process.

**Figure 6:**
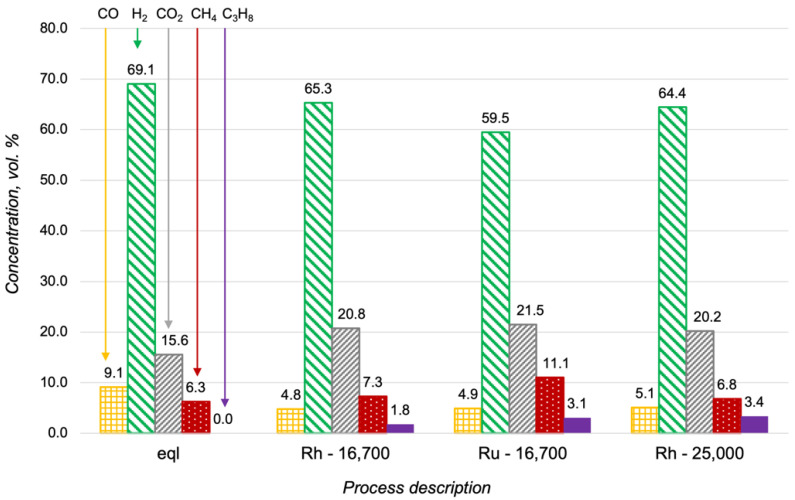
Product distribution in the propane steam reforming at 600 °C with H_2_O/C = 3 in a thermodynamic equilibrium (eql), over the Rh/CZA/FeCrAl at GHSV = 16,700 h^−1^ (Rh—16,700), over the Ru/CZA/FeCrAl at GHSV = 16,700 h^−1^ (Ru—16,700), and over the Rh/CZA/FeCrAl at GHSV = 25,000 h^−1^ (Rh—25,000).

Additional studies of the propane steam reforming at higher flow rates showed that the Rh/CZA/FeCrAl was more active than the Ru/CZA/FeCrAl (Figure 6). In particular, the concentration of the propane in the outlet gas mixture in the case of the Ru-based catalyst at 16,700 h^−1^ was approximately the same as the propane content in the reaction products over the Rh/CZA/FeCrAl at 25,000 h^−1^. According to the obtained results, the Rh/CZA/FeCrAl was chosen for the investigations of the oxidative steam reforming of propane.

### 3.3. Propane Oxidative Steam Reforming over the Pt- and Rh/CZA/FeCrAl

**Figure 7:**
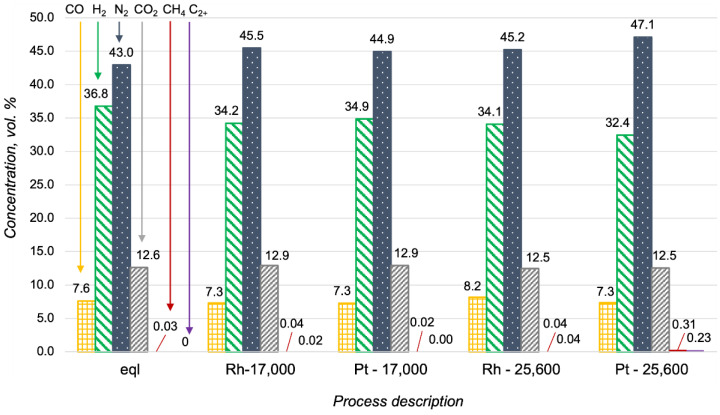
Product distribution in propane oxidative steam reforming at 700 °C with H_2_O/C = 2.5 and O_2_/C = 0.5 in a thermodynamic equilibrium (eql), over the Rh/CZA/FeCrAl and the Pt/CZA/FeCrAl at GHSV = 17,000 h^−1^ (“Rh—17,000” and “Pt—17,000”, respectively), and over the Rh/CZA/FeCrAl and the Pt/CZA/FeCrAl at GHSV = 25,600 h^−1^ (“Rh—25,600” and “Pt—25,600”, respectively).

The high activity of Pt-based catalysts in oxidative processes is well known [23,24]. It was the reason for the investigation of the Pt/CZA/FeCrAl in propane oxidative steam reforming, despite the obtained proof of its low activity in propane steam reforming. In Figure 7, the data of the Pt- and Rh/CZA/FeCrAl activities in propane oxidative steam reforming were compared with the product distribution in a thermodynamic equilibrium. The catalysts demonstrated high activities in propane oxidative steam reforming at 700 °C, with H_2_O/C = 2.5, O_2_/C = 0.5, and GHSV = 17,000 h^−1^, and the product distributions were quite close to equilibrium in both cases. At higher reagent flow rates, the concentration of hydrogen in the outlet gas mixtures decreased simultaneously with the increasing C_2+_-hydrocarbons and methane content (in the case of the Pt/CZA/FeCrAl). In general, the activity of the Pt/CZA/FeCrAl in propane oxidative steam reforming at GHSV = 25,600 h^−1^ was significantly lower than the Rh/CZA/FeCrAl activity.

**Figure 8:**
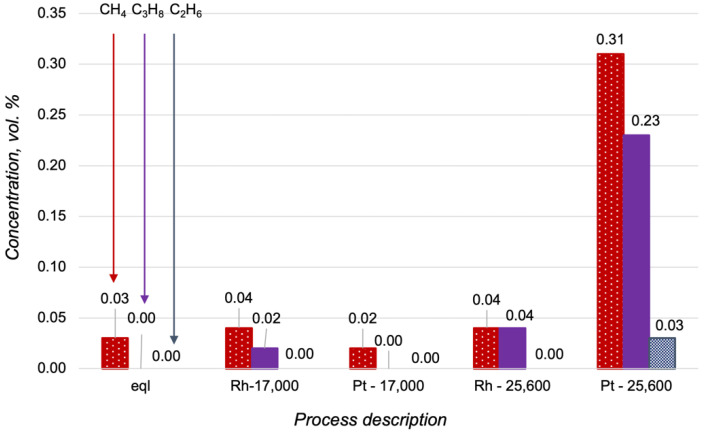
Hydrocarbon content in the products of the propane oxidative steam reforming at 700 °C, with H_2_O/C = 2.5 O_2_/C = 0.5 in a thermodynamic equilibrium (eql), over the Rh/CZA/FeCrAl and the Pt/CZA/FeCrAl at GHSV = 17,000 h^−1^ (“Rh—17,000” and “Pt—17,000”, respectively), and over the Rh/CZA/FeCrAl and the Pt/CZA/FeCrAl at GHSV = 25,600 h^−1^ (“Rh—25,600” and “Pt—25,600”, respectively).

More detailed information about hydrocarbon content in the reforming products is presented in Figure 8. Interestingly, C_2+_-hydrocarbons were not observed in the outlet gas mixture obtained over the Pt/CZA/FeCrAl at 17,000 h^−1^. This could be a sign of the high rate of propane oxidation in the initial zone of the Pt-based catalyst. The increasing of reagents flow rate led to decreases in the contact times between the reagents and the Pt active sites in the initial zone of the catalyst, and therefore, according to the low activity of Pt/CZA/FeCrAl in the steam reforming, some quantities of propane were not converted. The Rh/CZA/FeCrAl showed stable activity at 17,000 h^−1^, which did not decrease at 25,600 h^−1^.

### 3.4. n-Butane Reforming over the Rh/CZA/FeCrAl

The investigation of the propane steam and oxidative steam reforming showed that among investigated structured catalysts, the Rh/CZA/FeCrAl had the best efficiency. In commercial LPG compositions, there are hydrocarbons heavier than propane, such as butane. According to the literature data [25], the problem of carbon formation in hydrocarbons reforming can be associated with hydrocarbon chain length because of differences in the intermediate species structure and content. The next step of the study was to approve the Rh/CZA/FeCrAl properties in the n-butane reforming.

**Figure 9:**
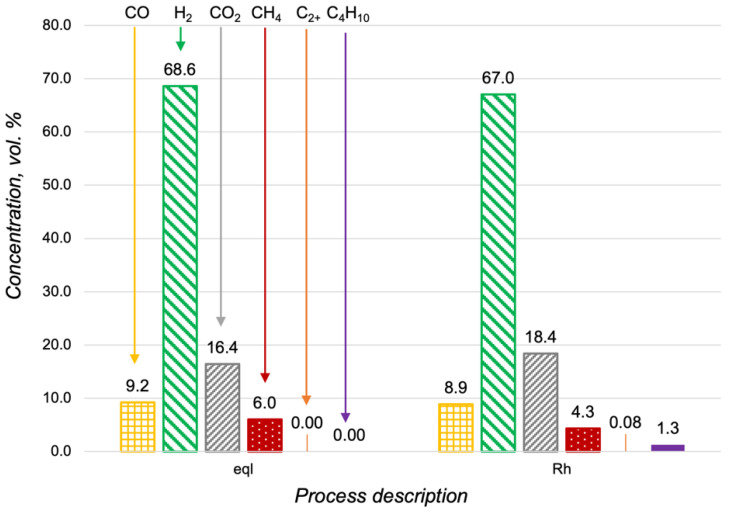
Hydrocarbon content in the products of the n-butane steam reforming at 600 °C, with H_2_O/C = 3 and O_2_/C = 0.5 in a thermodynamic equilibrium (eql) and over the Rh/CZA/FeCrAl at GHSV = 8125 h^−1^.

The process of the n-butane steam reforming was carried out at 600 °C, with H_2_O/C = 3 and GHSV = 8125 h^−1^. In Figure 9, the results of the Rh/CZA/FeCrAl activity investigation are presented. As seen in a comparison of Figure 5 and Figure 9, the content of unconverted fuel in the products of the n-butane steam reforming was higher than that in the case of propane. In general, it can be concluded that the Rh/CZA/FeCrAl demonstrated a rather high activity in both propane and n-butane steam reforming.

The results of our previous study of liquid fuels reforming over a Rh-based structured catalyst showed that carbon fibers had formed on the catalyst surface [17,26]. It was concluded that carbon formation was associated with the content of aromatic compounds in the fuel composition. To improve on that conclusion, we investigated the Rh/CZA/FeCrAl after the propane and n-butane steam reforming using the TPO method. According to the obtained data, in both cases, no carbon fibers had formed on the catalyst surface.

**Figure 10:**
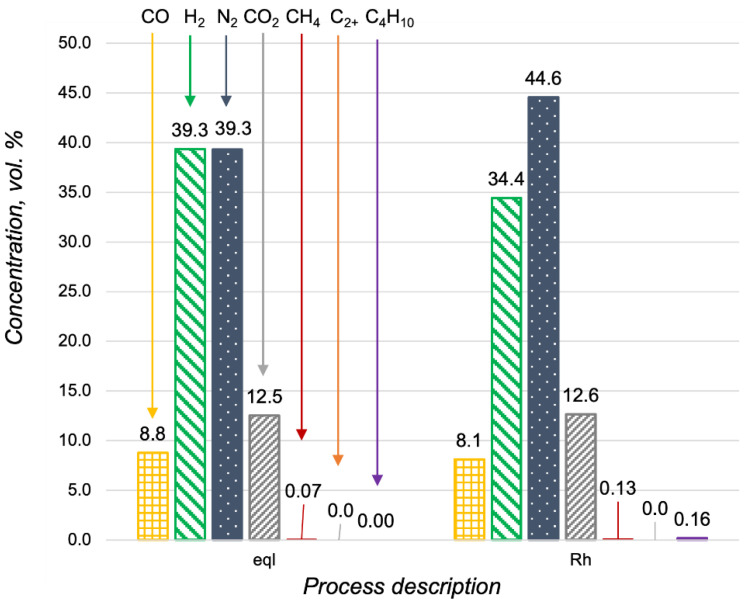
Product distribution in the n-butane oxidative steam reforming at 700 °C, with H_2_O/C = 2.5 O_2_/C = 0.5 in a thermodynamic equilibrium (eql) and over the Rh/CZA/FeCrAl at GHSV = 16,600 h^−1^.

The catalytic activity of the Rh/CZA/FeCrAl was measured in n-butane oxidative steam reforming at 700 °C, with H_2_O/C = 2.5, O_2_/C = 0.5, and GHSV = 16,600 h^−1^. In Figure 10, the composition of the reforming products was compared with the product distribution in a thermodynamic equilibrium. According to the obtained data, in the catalytic reforming, the n-butane was nearly totally converted. In the case of the propane oxidative steam reforming, C_2+_-hydrocarbons were not observed in the outlet gas mixture.

The measurements of the Rh/CZA/FeCrAl catalytic activity showed that the catalyst was highly efficient in the processes of propane and n-butane oxidative steam reforming, and in both cases, the fuel was converted to a hydrogen-rich gas mixture. It was especially important that in the steam and oxidative steam reforming of the fuels, carbon formation was not observed. It can be concluded that the Rh/CZA/FeCrAl is a perspective catalyst for LPG reforming with the goal of obtaining a syngas.

### 3.5. Mathematical Modelling

For processes such as the steam and oxidative steam reforming of propane, heat management plays a crucial role. The idea of material design was intended to improve the catalysts’ ability to heat transfer for enhancing the slow endothermic reactions of the steam reforming. To quantify the effect, we performed the mathematical modelling of the propane steam and oxidative steam reforming over the Pt- and Rh-based catalysts.

We used the model in the 2D axisymmetric geometry developed earlier for the diesel ATR process over a Rh-based catalyst [27,28]. The catalytic module was considered as a porous medium. The model took into account the velocity field and pressure distribution (Navier–Stokes equations for the free medium and Brinkman equations for the porous medium of the catalytic module), mass balance (convective and diffusion transfers and reaction source), and temperature distribution for the gas and catalyst, as follows:

(1) $$\nabla \cdot j_{i}+\rho \left(u\cdot \nabla \right)\omega_{i}=R_{i},$$

(2) $$j_{i}=-\left(\rho D_{i}^{m}\nabla \omega_{i}+\rho \omega_{i}D_{i}^{m}\frac{\nabla M_{n}}{M_{n}}-\rho \omega_{i}\sum_{k}\frac{M_{i}}{M_{n}}D_{k}^{m}\nabla x_{k}\right),\;\mathrm{and}$$

(3) $$D_{i}^{m}=\frac{1-\omega_{i}}{\sum_{k\neq i}\frac{c_{k}}{D_{ik}}}.$$

The velocity field (*u*) and pressure (*p*) were set by the “Brinkman Equations” interface shown in (4) and (5).

(4) $$0\;=\;\nabla \cdot [-pI+\mu \frac{1}{\varepsilon_{p}}\left(\nabla u+{\left(\nabla u\right)}^{T}\right)-\frac{2\mu }{3\varepsilon_{p}}\left(\nabla \cdot u\right)I]-u\left(\mu K^{-1}+\frac{Q_{m}}{\epsilon_{p}^{2}}\right)$$

(5) $$\nabla \cdot \left(\rho u\right)=Q_{m}$$

The heat transfer was assigned through the “Heat Transfer in Porous Media” for the gas and catalyst phases interface (i.e., it was supposed to be the quasi-homogeneous model, and so the gas and catalyst temperatures were described by the same variable).

(6) $$\rho C_{P}u\cdot \nabla T-\nabla \cdot \left(\left(1-\varepsilon_{p}\right)\lambda_{c}+\varepsilon_{p}\lambda \right)\nabla T=Q$$

The chemical transformations were assumed to take place according to the following set of reactions:

(7) $$\mathrm{CO}+H_{2}O↔{\mathrm{CO}}_{2}+H_{2}\;\Delta H_{298}=-41\;\mathrm{kJ}/\mathrm{mol}$$

(8) $${\mathrm{CH}}_{4}+H_{2}O↔\mathrm{CO}+3H_{2}\;\Delta H_{298}=206\;\mathrm{kJ}/\mathrm{mol}$$

(9) $$\mathrm{CO}+0.5O_{2}\;\to {\mathrm{CO}}_{2}\;\Delta H_{298}=-283\;\mathrm{kJ}/\mathrm{mol}$$

(10) $$H_{2}+0.5O_{2}\;\to H_{2}O\;\Delta H_{298}=-286\;\mathrm{kJ}/\mathrm{mol}$$

(11) $${\mathrm{CH}}_{4}+2O_{2}\to {\mathrm{CO}}_{2}+2H_{2}O\;\Delta H_{298}=-803\;\mathrm{kJ}/\mathrm{mol}$$

(12) $$C_{3}H_{8}+5O_{2}\to 3{\mathrm{CO}}_{2}+4H_{2}O\;\Delta H_{298}=-2045\;\mathrm{kJ}/\mathrm{mol}$$

(13) $$C_{3}H_{8}+3H_{2}O\to 3\mathrm{CO}+7H_{2}\;\Delta H_{298}=499\;\mathrm{kJ}/\mathrm{mol}$$

(14) $$C_{3}H_{8}+2H_{2}\to 3{\mathrm{CH}}_{4}\;\Delta H_{298}=-121\;\mathrm{kJ}/\mathrm{mol}$$

The inlet gas mixture was supposed to be evenly mixed and have a composition similar to that reported for the experiments above. The occurrence of homogeneous reactions in a free medium before and after the catalyst was neglected. The inlet mixture temperature was set at 600 °C for the steam reforming and 700 °C for the oxidative steam reforming, and the catalyst was located inside the hot box (outer cartridge walls) with a constant set temperature of 700 °C. All the modification calculations were compared with the reference case—the cylindrical catalytic module with a 9 mm radius and a 60 mm length. The volumes of the catalyst in all the modifications were the same as in the reference case, unless otherwise stated. COMSOL Multiphysics was used for the simulation.

The experimental data were used to adjust the kinetic parameters. The set of kinetic equations and values of kinetic parameters used are presented in Table 1 (the kinetic constants were used in an Arrhenius form). The concentrations are given in mole fractions. For the steam reforming process, only the reactions without oxygen were used (as in Equations (7), (8), (13) and (14)).

The modeling results of the propane SR (Table 2) and OSR (Table 3) are presented below. Generally, it is seen that the model can well describe both the SR and OSR of propane over the Pt/CZA/FeCrAl and the Rh/CZA/FeCrAl catalysts at various temperatures and flow rates. The high performance of both catalyst in the oxidative steam reforming (despite the low performance of the Pt/CZA/FeCrAl in the SR of propane) is associated with the high heat conductivity of the FeCrAl support and the effective heat transfer from the exothermal total oxidation of the propane in the front zone to the endothermal propane steam reforming in the end zone of the catalyst bed.

## 4. Discussion

The efficiency of the studied catalysts was compared with the literature data on the activity of catalysts based on Rh and Pt. The properties of granular Pt/CeO_2_ and Rh/Al_2_O_3_ catalysts in propane OSR were studied in [29] and [30]. The authors of [29] studied the properties of Pt/CeO_2_ pellets and two structured Pt/CeO_2_ catalysts: Pt/CeO_2_/cordierite and a self-structured Pt/CeO_2_ catalyst. A comparison of the results showed that the activity of both self-structured Pt/CeO_2_ and Pt/CeO_2_ deposited on a cordierite monolith in propane OSR were higher than the activity of a granular Pt/CeO_2_ catalyst in the same conditions. In [30], the catalyst design was similar to the catalysts presented in this article, and Rh/Al_2_O_3_ deposited on a rectangular structural block of FeCrAl foil was studied. The Rh/CZA/FeCrAl and Pt/CZA/FeCrAl catalysts studied in this work outperformed the mentioned catalysts in syngas productivity.

Solid oxide fuel cells (SOFC) are one of the prospective types of power generators. The key benefits of SOFC are its wide range of working temperatures and flexibility in fuel composition. Fuels applicable for SOFC can contain inert components such as N_2_ and CO_2_ and up to 20% of CO [31], 10% of CH_4_ [32], and 1.8% of C_2+_-hydrocarbons [33]. In Table 4, the compositions of the syngases obtained over a M/CZA/FeCrAl in the investigated processes were compared with the requirements for the SOFC’s fuel.

As seen in Table 4, in general, the syngases obtained over the Rh/CZA/FeCrAl in the propane and n-butane reforming processes are applicable for SOFCs. The Pt-based catalyst is also suitable for propane’s conversion to syngas in oxidative steam reforming conditions.

## 5. Conclusions

Highly dispersed composite structured M/Ce_0.75_Zr_0.25_O_2_/Al_2_O_3_/FeCrAl (M = Pt, Rh, and Ru) catalysts were prepared and tested in the steam and oxidative steam reforming of propane. The preparation process was monitored by XRD and TEM at different stages. It was shown that the suggested preparation procedures allowed us to obtain noble metal particles with an average size of 2–3 nm.

Rh/Ce_0.75_Zr_0.25_O_2_/Al_2_O_3_/FeCrAl outperformed other catalysts in propane reforming in terms of activity and syngas productivity. The syngases obtained in the propane steam reforming over Rh- and Ru/Ce_0.75_Zr_0.25_O_2_/Al_2_O_3_/FeCrAl at 600 °C and GHSV = 8300 h^−1^ contained 65.2 and 62.4 vol.% of H_2_, respectively, and can be used as fuel for solid oxide fuel cells. In the oxidative steam reforming of propane at 700 °C and GHSV= 17,000 h^−1^, the activities of Rh- and Pt-based were similar and the compositions of the outlet gas mixtures were quite close to equilibrium in both cases. Increasing the reagent flow rate to 25,600 h^−1^ showed the stability of the Rh/Ce_0.75_Zr_0.25_O_2_/Al_2_O_3_/FeCrAl performance, whereas the Pt/Ce_0.75_Zr_0.25_O_2_/Al_2_O_3_/FeCrAl activity decreased. The activity of the Rh-based catalyst was also proven in n-butane steam and oxidative steam reforming.

A mathematical model that included the velocity field, mass balance, pressure, and temperature distribution, as well as the reaction kinetics, was suggested for propane steam and oxidative steam reforming over the Pt- and Rh/Ce_0.75_Zr_0.25_O_2_/Al_2_O_3_/FeCrAl catalysts. The model well described the experimental results.

## Figures and Tables

**Table 1:** Kinetic parameters for Equations (7)–(14).

Catalyst	Reaction	Reaction Rate	k_0_, mol/(m^3^·s)	E, kJ/mol
Pt/CZA/FeCrAl	7	$k_{7}\left(c_{CO}c_{H_{2}O}-\frac{c_{CO_{2}}c_{H_{2}}}{\exp\left(\frac{4577.8}{T}-4.33\right)}\right)$	1.3 × 10^6^	41.5
	8	$k_{8}\left(c_{CH_{4}}c_{H_{2}O}-\frac{c_{CO}c_{H_{2}}^{3}}{\exp\left(-\frac{26800}{T}+29.8\right)}\right)$	5.3 × 10^5^	50
	9	$k_{9}c_{CO}c_{O_{2}}$	4.0 × 10^8^	80
	10	$k_{10}c_{H_{2}}c_{O_{2}}$	2.0 × 10^6^	40
	11	$k_{11}c_{CH_{4}}c_{O_{2}}^{0.5}$	8.0 × 10^6^	100
	12	$k_{12}c_{C_{3}H_{8}}c_{O_{2}}$	4.9 × 10^8^	100
	13	$k_{13}c_{C_{3}H_{8}}c_{H_{2}O}^{0.1}$	7.0 × 10^11^	180
	14	$k_{14}c_{C_{3}H_{8}}c_{H_{2}}$	2.0 × 10^7^	100
Rh/CZA/FeCrAl	7	$k_{7}\left(c_{CO}c_{H_{2}O}-\frac{c_{CO_{2}}c_{H_{2}}}{\exp\left(\frac{4577.8}{T}-4.33\right)}\right)$	1.0 × 10^6^	41.5
	8	$k_{8}\left(c_{CH_{4}}c_{H_{2}O}-\frac{c_{CO}c_{H_{2}}^{3}}{\exp\left(-\frac{26800}{T}+29.8\right)}\right)$	3.6 × 10^3^	10
	9	$k_{9}c_{CO}c_{O_{2}}$	4.0 × 10^8^	80
	10	$k_{10}c_{H_{2}}c_{O_{2}}$	2.0 × 10^6^	40
	11	$k_{11}c_{CH_{4}}c_{O_{2}}^{0.5}$	8.0 × 10^6^	100
	12	$k_{12}c_{C_{3}H_{8}}c_{O_{2}}$	1.0 × 10^8^	100
	13	$k_{13}c_{C_{3}H_{8}}c_{H_{2}O}^{3}$	2.9 × 10^6^	40
	14	$k_{14}c_{C_{3}H_{8}}c_{H_{2}}^{2}$	1.6 × 10^3^	10

**Table 2:** Comparison of the modelling results and experiments for the propane SR.

Catalyst		Concentration on Dry Basis, vol.%				
		CO	CO_2_	CH_4_	H_2_	C_3_H_8_
Pt/CZA/FeCrAl	Exp	0.6	11.2	0.2	38.7	49.2
	Model	0.7	10.9	0.3	39.1	47.8
Rh/CZA/FeCrAl	Exp	6.6	19.3	8.7	65.2	0.2
	Model	6.5	17.4	7.2	68.3	0.25

**Table 3:** Comparison of the modelling results and experiments for the propane OSR.

Catalyst		GHSV, h^−1^	Concentration on Dry Basis, vol.%					
			CO	CO_2_	CH_4_	H_2_	C_3_H_8_	N_2_
Pt/CZA/FeCrAl	Exp	20,000	7.3	12.8	0.0	34.8	0.00	45.1
	Model	20,000	7.4	12.4	0.1	34.9	0.21	45.0
	Exp	30,000	7.4	12.5	0.3	32.2	0.28	47.3
	Model	30,000	7.8	12.2	0.1	34.9	0.17	44.9
Rh/CZA/FeCrAl	Exp	20,000	6.4	13.6	0.0	34.9	45.0	0.02
	Model	20,000	7.7	12.4	0.1	35.8	44.1	0.03
	Exp	30,000	6.7	13.5	0.0	34.6	45.2	0.05
	Model	30,000	8.2	12.0	0.1	35.3	44.5	0.06

**Table 4:** Comparison of the requirements for the SOFCs fuel and the compositions of the syngases obtained over the M/CZA/FeCrAl.

			Concentration on Dry Basis, vol.%			
			H_2_	CO	CH_4_	C_2+_
SOFC’s Fuel Requirements			-	<20	<10	<1.8
Process	Catalyst	GHSV, h^−1^	H_2_	CO	CH_4_	C_2+_
C_3_H_8_ SR	Rh/CZA/FeCrAl	8300	65.2	6.6	8.7	0.2
		16,700	65.3	4.8	7.3	1.8
		25,000	64.4	5.1	6.8	3.4
	Ru/CZA/FeCrAl	8300	62.3	7.1	8.8	0.7
		16,700	59.5	4.9	11.1	3.2
C_3_H_8_ OSR	Rh/CZA/FeCrAl	17,000	34.2	7.3	0.04	0.04
		25,600	34.1	8.2	0.04	0.02
	Pt/CZA/FeCrAl	17,000	34.9	7.3	0.02	0.00
		25,600	32.4	7.3	0.31	0.23
n-C_4_H_10_ SR	Rh/CZA/FeCrAl	8125	67.0	8.9	4.3	1.38
n-C_4_H_10_ OSR		16,600	34.4	12.6	0.13	0.16

## Data Availability

Not applicable.
